# In this month’s Bulletin

**DOI:** 10.2471/BLT.17.001217

**Published:** 2017-12-01

In the editorial section, Patrick L Osewe (794) discusses options for financing pandemic preparedness. Dermot Maher & Nathan Ford (795) propose that the public health research agenda is informed by questions arising during guideline development. 

Tatum Anderson (798–799) reports on measures taken by several countries to prevent skin cancer by restricting the use of sunbeds. Fatima Suleman talks to Fiona Fleck (800–801) about efforts to make affordable medicines available, particularly in South Africa. 

## Belgium

### Returning travellers exposed to Zika.

Ralph Huits et al. (802–810) quantify Zika virus in semen samples.

## Botswana, Burkina Faso, Burundi, Cameroon, Côte d’Ivoire, Democratic Republic of the Congo, Egypt, Ethiopia, Kenya, Lybia, Mali, Morocco, Niger, Nigeria, Rwanda, Senegal, South Africa, Sudan, Togo, Uganda, United Republic of Tanzania

### Occupational infectious hazards

Asa Auta et al. (831–841) collate the evidence on health care workers’ exposures to body fluids. 

## India

### An elusive cause for endemic nephropathy

Praveen Gadde et al. (848–849) chart a difficult course of disease definition and prevention.

## Sierra Leone

### Nurses providing mental health services

Stania Kamara et al. (842–847) describe mental health care provided during the Ebola outbreak. 

## United Republic of Tanzania

### Getting mothers to care providers

Claudia Hanson et al. (811–821) document geographical inequalities in access to services.

## Global

### Improving food quality

Lirije Hyseni et al. (822–830) review the evidence for interventions designed to reduce trans-fat.

### Improving lives of children left behind

Catherine Jan et al. (850–852) discuss health trends of migrant workers’ children.

